# Efficacy of iron supplementation on physical capacity in non-anaemic iron-deficient individuals: protocol for an individual patient data meta-analysis

**DOI:** 10.1186/s13643-024-02559-4

**Published:** 2024-07-15

**Authors:** Cory Dugan, Peter Peeling, Richard Burden, Toby Richards

**Affiliations:** 1 School of Human Sciences, University of Western Australia, Perth, Australia; 2 National Centre for Sport and Exercise Medicine, School of Sport, Exercise and Health Sciences, Loughborough University, Loughborough, UK; 3 Division of Surgery, University of Western Australia, Perth, Australia

**Keywords:** Iron deficiency, Non-anaemia, Iron supplementation, Physical capacity, Quality of life, Individual patient data

## Abstract

**Background:**

A deficiency in iron stores is associated with various adverse health complications, which, if left untreated, can progress to states of anaemia, whereby there is significant detriment to an individual’s work capacity and quality of life due to compromised erythropoiesis. The most common methods employed to treat an iron deficiency include oral iron supplementation and, in persistent and/or unresponsive cases, intravenous iron therapy. The efficacy of these treatments, particularly in states of iron deficiency without anaemia, is equivocal. Indeed, both randomised control trials and aggregate data meta-analyses have produced conflicting evidence. Therefore, this study aims to assess the efficacy of both oral and intravenous iron supplementation on physical capacity, quality of life, and fatigue scores in iron-deficient non-anaemic individuals using individual patient data (IPD) meta-analysis techniques.

**Methods:**

All potential studies, irrespective of design, will be sourced through systematic searches on the following databases: Cochrane Central Register of Controlled Trials, MEDLINE Ovid, Embase Ovid, Web of Science: Science Citation Index Expanded, Web of Science: Conference Proceedings Citation Index-Science, ClinicalTrials.gov, and World Health Organization (WHO) International Clinical Trials Registry Platform. Individual patient data from all available trials will be included and subsequently analysed in a two-stage approach. Predetermined subgroup and sensitivity analyses will be employed to further explain results.

**Discussion:**

The significance of this IPD meta-analysis is one of consolidating a clear consensus to better inform iron-deficient individuals of the physiological response associated with iron supplementation. The IPD approach, to the best of our knowledge, is novel for this research topic. As such, the findings will significantly contribute to the current body of evidence.

**Systematic review registration:**

PROSPERO CRD42020191739.

**Supplementary Information:**

The online version contains supplementary material available at 10.1186/s13643-024-02559-4.

## Introduction

The micronutrient, iron, has an essential role in the transport and storage of oxygen as well as cellular and mitochondrial respiration [1, 2]. Further, iron is vital for healthy immune function in conjunction with cell growth [1]. Consequently, both excessive and scarce iron levels are associated with various adverse health complications [1, 3]. In athletic populations, previous literature has categorised ID into three stages of severity, each with varying impacts on exercise performance [4]. These include the following:

1. Iron depletion (iron deficiency nonanaemia stage I): Iron stores in the bone marrow, liver, and spleen are depleted (serum ferritin: < 35 μg/L; Hb: > 115 g/dL; transferrin saturation: > 16%).
2. Iron-deficient erythropoiesis (iron deficiency nonanaemia stage II): Erythropoiesis diminishes as the iron supply to the erythroid marrow is reduced (serum ferritin: < 20 μg/L; Hb: > 115 g/dL; transferrin saturation: < 16%).
3. Iron-deficient anaemia: Haemoglobin production falls, resulting in anaemia (serum ferritin: < 12 μg/L; Hb: < 115 g/L; transferrin saturation: < 16%).

Iron deficiency (ID) is the most common nutrient deficiency globally, affecting approximately one third of the population (2 billion people) [5, 6]. Iron deficiency can present itself through an array of symptoms or can be asymptomatic [7]. The most commonly reported symptoms include physical and mental fatigue, brain fog (the inability to think clearly), shortness of breath, heart palpitations, alopecia (hair loss), and pica (the desire to eat non-foods such as dirt) [7–9]. Further to this, evidence in mouse models suggest that iron deficiency negatively affects physical endurance, being responsible for decreases in mitochondrial complex I activity of oxidative skeletal muscle [10]. Recent research in human participants has also highlighted impaired skeletal muscle metabolism in the presence of iron deficiency; however, no impairment of muscle oxidative phosphorylation was observed [11]. Rather, disturbed blood lactate kinetics were demonstrated, appearing to promote a shift towards anaerobic glycolysis, which could explain, in part, the negative impact seen on aerobic capacity and fatigue scores [11–13].

Without treatment, ID can progress in severity, resulting in states of iron deficiency anaemia (IDA), characterised by exhausted iron stores, a reduction in erythrocyte production, and a decrease in haemoglobin concentration [14]. Consequently, oxygen-carrying capacity to the working muscle is compromised, inevitably decreasing V̇O_2max_ [15]. Such states are linked to even greater declines in work capacity and quality of life [5, 6, 16–22].

The strong causal relationship between IDA and exercise capacity has brought about a plethora of research to determine the best strategies to replenish iron stores. To date, the most common iron supplementation methods include oral supplementation and, in persistent, severe, and/or unresponsive cases, intravenous (IV) iron therapy [23–25].

### Iron supplementation methods

#### Oral iron therapy

Oral iron therapy is generally consumed in either tablet or liquid preparations, commonly available in ferrous or ferric forms. Typically, only ~ 10% of intestinal iron is absorbed [26], meaning the restoration of iron levels via the gut can take extended periods of time [27]. Despite this, research has demonstrated modest efficacy in iron store replenishment, with increases of serum ferritin ranging between 40 and 80% in healthy populations [28, 29] following recommended daily doses of ~ 100–200 mg of elemental iron per day over a 6–8-week period [23, 25]. Regardless, it should be noted that oral iron therapy has been shown to be ineffective at replenishing iron stores in cases of ID caused by chronic inflammation (functional ID). This is due to a reduced dietary iron absorption and failure of cellular iron export into circulation in the presence of inflammation, thus reducing iron availability despite the presence of adequate iron stores [30]. Consequently, alternative methods of iron repletion should be considered here.

#### Intravenous iron therapy

Over the last decade, the use of intravenous (IV) iron therapy has increased considerably due to improvement in availability as well as increased safety of modern preparations [30]. Modern IV iron preparations are made up of an iron core (ferric hydroxide particles) within a carbohydrate shell for the purposes of delaying iron release [23]. Unlike oral iron supplementation, IV iron therapy restores iron levels within approximately 24 h [31], with direct injection of iron into the circulation allowing for absorption restrictions at the gut to be bypassed [32]. Such a prospect allows for rapid increases in iron status, with research in healthy populations reporting 200–400% increases in ferritin levels from a 300 to 550 mg dose of iron [28, 29, 32, 33]. Although previous reviews have demonstrated no association between IV iron therapy and serious adverse events [34], current guidelines still only recommend its use in severe cases, where appropriate equipment and staff can be utilised to avoid and or manage hypersensitivity reactions [7].

#### Efficacy of oral and IV iron supplementation on ID individuals

In states of IDA, whereby oxygen-carrying capacity is compromised due to decreased haemoglobin concentrations, the benefits of iron therapy on physical capacity have been well demonstrated [35, 36]. This is simply due to the correction of haemoglobin concentration allowing for an increased oxygen carrying capacity. In contrast, states of ID without anaemia (IDNA), whereby oxygen-carrying capacity is not compromised, the evidence is equivocal. Studies specifically utilising oral supplementation routes have seen both increases [37–42] and no change [29, 43, 44] in physical capacity. Similar ambiguity has been documented in studies involving IV iron therapy, where increases [29] and no change [32, 33] to physical capacity are also shown. Of note, it has been suggested that the divergence of findings is due to major statistical and methodological heterogeneity across studies [45] coupled with the challenge of separating the effects of ID from the consequences of potential anaemia [11].

The aforementioned divergence has been highlighted by several systematic reviews and meta-analyses [45–48]. As with the ambivalence found in the clinical trials, differing conclusions also exist across the review papers. One such review, conducted by Rubeor and colleagues [48], involved 12 studies (9 oral, 1 IV, and 2 intramuscular interventions) with 283 participants, where half of the included studies (6 studies; 146 participants) showed performance improvements in IDNA athletes that were treated. Of note, it should be mentioned that all of these studies used a ferritin level cut-off of ≤ 20 μg/L for treatment. Therefore, it was concluded that iron supplementation could improve physical capacity when serum ferritin is ≤ 20 μg/L [48]. It should be noted that this study did not define non-anaemia, with the inclusion criteria stating ‘participants defined by the study authors as having IDNA or low ferritin without anaemia’ [48].These finding has been both corroborated [46] and refuted [47] by other meta-analyses, and therefore, it can be concluded that conventional meta-analysis techniques (i.e. aggregate data) have failed to provide clear consensus regarding the efficacy of iron supplementation, both oral and IV, in IDNA individuals [45].

### Heterogeneity of literature

A recent Cochrane review [45], also investigating the efficacy of iron supplementation (IV only) in IDNA individuals, explained that substantial heterogeneity significantly impacted the outcomes, primarily due to the aforementioned differences in current research protocols (i.e. different definitions of iron deficiency, different participant characteristics, inconsistent administration routes, and varying research protocols), which consequently results in analyses of limited clinical significance due to low quality evidence [45]. Furthermore, very low-quality evidence from the included studies in relation to V̇O_2max_ and quality-of-life measures meant that appropriate analysis could not be conducted accurately. As such, different methods (such as meta-analysis by individual patient data) should be explored to control for such heterogeneity.

#### Serum ferritin thresholds for defining iron deficiency

Due to the and lack of consensus for serum ferritin values to define iron deficiency across studies, conventional meta-analysis techniques may not be effective on the basis that the studies are not comparable. The current serum ferritin thresholds for defining iron deficiency (serum ferritin < 15 µg/L) determined by the World Health Organization (WHO) are based on expert opinion and are supported by a low to very low certainty of evidence [49, 50]. The WHO have acknowledged their guidelines, and ferritin cut-off values are based on expert opinion, not on published data [51]. Due to this, higher threshold recommendations are commonly adopted to increase the sensitivity of ferritin to detect iron deficiency (for example the Australian Medical Association defines ID as a serum ferritin < 30 µg/L). Although these higher ferritin thresholds have increased the sensitivity to detect iron deficiency, there is still little to no evidence justifying them [52].

Despite being widely accepted, the rationale concerning the quantitative values lack empirical support and are still being based upon expert opinion or serum ferritin distributions of varying populations. Physiologically based ferritin thresholds for iron deficiency in conjunction with other blood markers (such as transferrin saturation, soluble transferrin receptor, hepcidin concentration, and haemoglobin concentration) would allow for better informed research [49, 52]. For instance, Mei and colleagues [49] aimed to identify the ferritin concentration at which soluble transferrin receptor concentration rises and haemoglobin concentration begins to decline in children and nonpregnant women. The association between the two independent indicators of iron-deficient erythropoiesis (i.e. haemoglobin and soluble transferrin receptor concentration) identified ferritin concentration thresholds of ~ 20 μg/L and ~ 25 μg/L to define iron deficiency in children and nonpregnant women, respectively.

### The need for meta-analysis of individual patient data

An individual patient data (IPD) meta-analysis, which summates the raw data from relevant studies, would allow for the standardisation of the inclusion criteria, thus enabling the comparison between studies with a single (physiologically derived) ferritin cut-off value for the definition of iron deficiency [53]. Resultantly, subject- and study-level sources of heterogeneity are taken into account, which may assist in exploring effect modification as well as adjusting for confounding variables [54]. For this reason, the IPD meta-analysis methodology has become increasingly common and is regarded as a ‘gold standard’ meta-analysis compared to conventional meta-analysis techniques [55].

### Aim

Overall, when considering cases of extreme heterogeneity in estimates of relative treatment effect, a weighted average may no longer be informative [54]. Consequently, synthesising aggregate data may be ineffective and produce conflicting results (as evidenced above), suggesting that alternative approaches should be considered. For this reason, the aim of the present study is to assess, using IPD meta-analysis methodology, the efficacy of iron supplementation (both oral and IV) on physical capacity, quality of life, and fatigue scores in IDNA individuals.

## Methods

An IPD meta-analysis will be conducted in accordance with the recommendations of the Preferred Reporting Items for Systematic Reviews and Meta-Analyses-IPD statement [56] in conjunction with the Cochrane handbook [57]. This study was prospectively registered on PROSPERO (registration ID, CRD42020191739; Additional file 1). This IPD meta-analysis will be investigating the efficacy of iron repletion therapies (both oral and IV) on physical capacity, fatigue, and quality of life in non-anaemic iron-deficient (IDNA) individuals.

### Systematic review to identify eligible papers

#### Types of studies

There will be no restriction on the type of study design eligible for inclusion.

### Types of participants

Participants of both sexes (aged between 12 and 55) with functional or absolute non-anaemic iron deficiency will be included. Definitions of IDNA and IDA are as follows in accordance with previous research [4]:

1. Iron-deficient erythropoiesis (IDNA): Erythropoiesis diminishes as the iron supply to the erythroid marrow is reduced (serum ferritin: < 20 μg/L; Hb: > 115 g/dL; transferrin saturation: < 16%).
2. Iron-deficient anaemia (IDA): Haemoglobin production falls, resulting in anaemia (serum ferritin: < 12 μg/L; Hb: < 115 g/L; transferrin saturation: < 16%).

As previously stated, the current serum ferritin thresholds for iron deficiency determined by the WHO (serum ferritin < 15 μg/L) are based on expert opinion and are supported by a low to very low certainty of evidence [49, 50]. The higher threshold chosen is a conservative cut-off value based on previous physiologically mechanistic research [49, 52], rather than expert opinion or serum ferritin distributions of varying populations. Further, this value is in line with the findings of Rubeor and colleagues [48], where it was concluded that iron supplementation could improve physical capacity when serum ferritin is ≤ 20 μg/L [48].

Participants with haemochromatosis or other relating genetic diseases will be excluded from the analysis. Further, participants with medical conditions requiring hospitalisation will not be included (e.g. heart failure patients). Females who are pregnant or currently lactating will also not be included. Finally, studies involving animals will not be included.

#### Types of interventions

All iron therapy interventions will be considered. These include the following: (1) oral iron supplement, (2) intravenous iron therapy, and (3) intramuscular iron therapy.

#### Comparison treatments

Eligible comparison treatments will be as follows: (1) no intervention, (2) placebo in any form, and (3) other active therapies that could be considered standard care (for example diet manipulation).

#### Types of outcome measures

The primary outcome measure will be change in physical capacity measured at the end of follow-up, as defined by the trial authors. Secondary outcome measures include fatigue scores as well as quality-of-life measurements at the end of follow-up, irrespective of measurement method. Other outcome measures include total haemoglobin mass and blood markers relating to iron status (haemoglobin concentration, serum ferritin, transferrin saturation (%), serum iron, haematocrit, transferrin, total iron-binding capacity, and soluble transferrin receptor), measured at the end of follow-up.

### Search methods for identification of studies

A systematic review to identify eligible papers will be performed. The following databases will be searched in accordance with the Cochrane Handbook for Systematic Reviews of Interventions from inception to the present date [57]: Cochrane Central Register of Controlled Trials, MEDLINE Ovid, Embase Ovid, Web of Science: Science Citation Index Expanded, Web of Science: Conference Proceedings Citation Index-Science, ClinicalTrials.gov, and World Health Organization (WHO) International Clinical Trials Registry Platform. Full details for the search strategy are available in the supplementary material (Additional file 2).

**Fig. 1:**
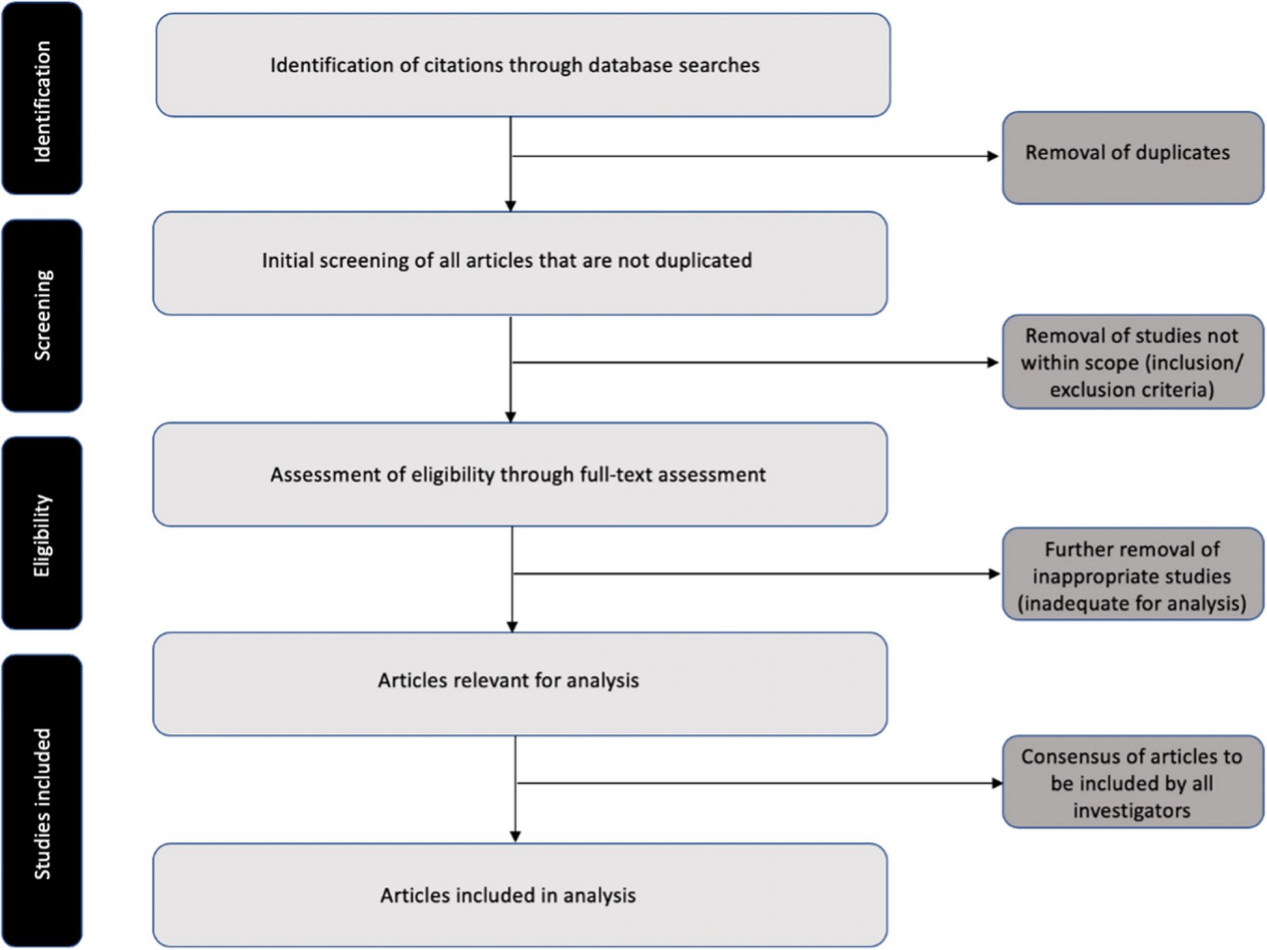
Study flow diagram following the Preferred Reporting Items for Systematic Reviews and Meta-Analysis criteria [57]

All citations identified through database searches will be imported into a single reference manager library. After the removal of duplicates, citations will be screened independently by a minimum of two trial authors in accordance with the predetermined eligibility criteria. Any disagreements will be resolved through the involvement of a third review author. Articles that are deemed eligible will be further examined via a full-text review. Once again, a third review author will be consulted regarding any disagreements. The remaining eligible articles to be included will be reviewed by all authors to attain consensus (Fig. 1).

### Collecting and aggregating individual patient data

#### Author collaboration request

Authors of eligible studies will be contacted and invited to collaborate on this project. A standardised email and database template will be created and sent to the contacted author for each study; however, databases will be accepted in all formats.

The process for contact is standardised and is described below:

1. Initial contact information will be attained and utilised from the published trials.
2. In the circumstance of no response after 2 weeks, a follow-up email will be sent.
3. If there is still no response after a further 2 weeks, then we will follow up with a phone call (if their phone number is available on their institutions website).
4. Failing that, social media will be utilised. Specifically, a personal message identical to the standardised email will be sent as an avenue for contact. The first social media platform used will be Twitter (X). In the case of no response after 7 days, messages will be sent on the following social media platforms in this order: ResearchGate, LinkedIn, Facebook, Instagram. Each 7 days without response, the next platform will be utilised. 
5. Finally, if there is still no response, the process will be repeated for the next listed author, until contact is made, or all authors have been exhausted. In the case of no contact, institutional libraries will be contacted in an attempt to attain any hard copy/printed versions of the required data.

#### Failed collaboration request procedure (combining IPD and aggregate data)

Low author response rates are expected, which could significantly impact successful data collection [58]. Indeed, despite the increased use of IPD meta-analysis techniques as well as changes to data sharing policy, rates of data retrieval for IPD meta-analysis have not improved [59]. To mitigate the foreseen impact, published practical guidance on obtaining and managing IPD will be employed [58, 60]. Furthermore, the combination of IPD and aggregate data will be considered if appropriate. Combining IPD and aggregate data in evidence synthesis has received little attention given the known challenges of obtaining IPD [61]. Indeed, very few proposed methods have been published [61, 62]. Interestingly, in spite of this, these methods are commonly used in the literature [61] and have been recommended as a way to examine the potential for publication bias, data availability bias and reviewer selection bias [63].

#### Initial data screening and IPD integrity

All received IPD will undergo screening to ensure it is acceptable to be included in the analysis. Specifically, data will be checked against the published paper for accuracy. Where appropriate, IPD will be used to recalculate descriptive characteristics of the included studies, to ensure consistency with the published paper. Should inconsistencies arise, authors will be contacted for clarification.

#### Data extraction and database creation

Anonymised datasets that complete initial screening and are deemed acceptable for inclusion in the analysis will be closely analysed by each author. Each author will individually extract relevant data using standardised forms. Upon consensus, data will be combined into a single CSV file that will be uploaded to R (Version 4.3.2). To ensure data security and confidentiality, all CSV files and R scripts will be stored on a secure, password-protected device, which is only accessible by authorised research personnel. This device will be owned by the University of Western Australia.

### Quality assessment (risk of bias)

The internal validity of the included studies will be assessed using the Cochrane Collaboration risk-of-bias tool 2 [57, 64, 65]. This tool assesses any potential bias associated with the following: the randomisation process, deviations from the intended interventions, missing outcome data, measurement of the outcome, and selection of the reported result [64].

### Data analysis

All statistical analyses will be performed in R (Version 4.3.2), and statistical significance will be considered with an alpha level of 0.05. Initially, the compiled database will be harmonised with unified coding. For instance, treatment conditions will be defined and coded according to the type of treatment in each study (oral iron supplement, intravenous iron therapy, intramuscular iron therapy, other).

#### Two-stage IPD meta-analysis

One-stage approaches have been favoured in the scientific literature—due to the capacity for complex modelling [66]. In this study, complex modelling is not necessary [67], and therefore, a two-stage IPD meta-analysis approach will be adopted for all outcome variables. Briefly, the two-stage IPD meta-analysis approach initially analyses the IPD from each study separately to determine the studies specific estimate (and CI) of the relative treatment effect [54]. Following this, conventional meta-analysis techniques will be utilised, whereby the effect estimates are combined in an appropriate model (outlined below) [54].

##### Stage 1

Appropriate statistical modelling to determine treatment effect will be used for each study based on the type of data obtained. Using the ‘lm’ function in R, linear regression models will be fitted to calculate estimated effect sizes (i.e. mean difference or standardised mean difference SMD) with their corresponding 95% CIs. When outcomes are measured on different scales across studies, standardised mean difference will be employed and converted to a common unit for consistency in accordance with the Cochrane handbook [57].

##### Stage 2

In stage 2, to calculate the pooled effect size of outcome variables, individual study effect estimates will be fitted into a random effects model (using the ‘rma’ function in the ‘metafor’ R package) to account for both within- and between-study variability. Studies will be weighted using the inverse variance method, whereby more weight is given to studies with lower variance. All analyses will be reported as a pooled effect size and its corresponding 95% CI in conjunction with measures of heterogeneity (described below). To visually demonstrate results, forest plots will be created (using the ‘forest’ function in the ‘metafor’ R package).

#### Heterogeneity

Due to heterogenous research protocols (i.e. different definitions of iron deficiency, different participant characteristics, inconsistent administration routes and varying research protocols), heterogeneity is expected in analyses. To explore heterogeneity, the chi-squared (*χ*^2^) test and the *I*^2^ statistic will be calculated using the ‘metafor’ package in R [68]. Considerable heterogeneity (i.e. *I*^2^ > 50%) will be further investigated through predetermined subgroup analysis.

#### Planned subgroup analysis

Where appropriate, subgroup analysis will be used to explore differences in iron supplementation efficacy between predetermined subsets of data (via the ‘mods’ argument in R). These subgroups include the following:

1. Sex (male vs. female)
2. Serum ferritin threshold (< 20 μg/L vs. < 12 μg/L)
3. Supplementation method (oral therapy vs. intravenous therapy vs. intramuscular therapy)
4. Supplementation dosage and duration (where appropriate)
5. Conflicts of interest (sponsored studies vs. non-sponsored studies)
6. Time to follow up

#### Planed sensitivity analysis

Sensitivity analyses will be used to determine the robustness of the findings. This analysis will exclude trials that were deemed at a high risk of bias, to assess any differences in findings. Another analysis will be conducted with IPD only, to investigate the robustness of including aggregate data where appropriate.

### Grading of evidence and summary of findings table

The overall quality of evidence will be graded using the Grading of Recommendations Assessment, Development and Evaluation (GRADE) methodology [57]. As outlined in the Cochrane handbook [57], this assessment grades the quality of evidence as either ‘very low’, ‘low’, ‘moderate’, or ‘high’ based on the following dimensions: (1) risk of bias, (2) inconsistency (unexplained heterogeneity of results), (3) indirectness of evidence, (4) imprecision of results, and (5) probability of publication bias. Where evidence quality is not deemed ‘high’, we will conclude that future research is likely to influence the estimate of effect in that outcome. A ‘Summary of findings table’ will be prepared using GRADEpro GDT software (GRADEpro GDT) to highlight evidence quality.

## Discussion

This will be the first IPD meta-analysis to investigate the efficacy of iron supplementation on physical capacity, quality of life and fatigue scores in IDNA individuals (both stages I and II). Conventional meta-analysis techniques (i.e. aggregate data) have been unable to provide clear consensus regarding the efficacy of iron supplementation on physical capacity in IDNA individuals [45–48, 69]. This study aims to contribute to the current body of evidence, through the reanalysis of current literature, to generate improved quality empirical evidence.

The IPD approach has been previously shown to substantially improve the quantity and quality of data whilst also allowing for the standardisation of outcomes across trials. This provides researchers with the ability to conduct more in-depth meta-analyses to generate robust results [55]. Furthermore, this approach has the capacity to differentiate between subject-level and study-level sources of heterogeneity in treatment effects. As a result, this will provide greater insight into effect modification due to the identification and adjustment of confounding variables [54]. For this reason, IPD meta-analyses have been termed the ‘gold standard’ for empirical evidence [53], with their use becoming increasingly common [53, 70].

In regard to the present study, the IPD approach will have significant advantages compared to other meta-analysis techniques. Firstly, the IPD approach allows for the homogeneity of outcome definitions across trials and the inclusion criteria (e.g. iron deficiency definition) [53], meaning the lack of clinical consensus in terminology (e.g. different definitions of iron deficiency) and the heterogenous trial protocols will not impact IPD analyses. The IPD approach will also permit more rigorous subgroup analyses, to investigate any differences in the efficacy of iron supplementation between varying populations. For instance, the relationship between iron supplementation efficacy and an individual’s serum ferritin concentration can be further explored, particularly given it has been previously hypothesised that iron supplementation could improve physical capacity when serum ferritin is ≤ 20 μg/L [48]. Finally, the IPD approach will provide the opportunity to check the integrity of trial IPD and resolve any queries with trial investigators. Considering the number of studies deemed to be at a high risk of bias (as defined by previous reviews [45–48]), this is important to address.

The aforementioned advantages of IPD meta-analyses will provide greater statistical power to determine the efficacy of iron supplementation on physical capacity in IDNA individuals. However, the IPD approach, as with all methodology, has numerous challenges, which should be considered. The major challenge with this approach is the susceptibility to selection or ‘availability bias’, due to the requirement of included studies to have IPD made available [59]. Due to the poor rates of data retrieval for IPD [59], the analysis may not be representative of the patient population, thus decreasing the quality of evidence. In addition to this, there is currently little guidance on how to conduct IPD meta-analysis. Indeed, a consensus of IPD meta-analyses techniques remains latent to the literature, which has resulted in wide variation in published methods [71]. Taken together, these challenges add to the already significant time investment for conducting an IPD meta-analysis. Overall, the benefits of an IPD approach supersede the required investment, as it is necessary to resolve the current ambiguity in the literature.

### Supplementary Information

Additional file 1. Prospero registration approval

Additional file 2. Draft search strategy.

Additional file 3. PRISMA-P 2015 Checklist.

## Data Availability

All data collection, analysis and sharing of this project will follow in accordance with the Cochrane handbook. Data sharing is not applicable to this protocol as no datasets were generated or analysed during the current study.

## Abbreviations

ID: Iron deficiency
IDA: Iron deficiency anaemia
IDNA: Iron deficiency non-anaemia
IPD: Individual patient data
IV: Intravenous
MD: Mean difference
SMD: Standardised mean difference
WHO: World Health Organization
