# Supplementary material for: Efficacy of iron supplementation on physical capacity in non-anaemic iron-deficient individuals: protocol for an individual patient data meta-analysis
Source: Syst Rev. 2024 Jul 15;13:182. doi: 10.1186/s13643-024-02559-4 (PMC11247796; doi:10.1186/s13643-024-02559-4)
Supplement: Supplementary file 1 — Additional file 1. Prospero registration approval [file 13643_2024_2559_MOESM1_ESM.pdf]

Efficacy of iron supplementation on physical capacity in non-anaemic iron deficient individuals: protocol for an individual patient data meta-analysis (IPMA)

Review methods were amended after registration. Please see the revision notes and previous versions for detail.

Citation

Cory Dugan, Toby Richards, Peter Peeling, Giulianna D'Aulerio, Lawrence Best. Efficacy of iron supplementation on physical capacity in non-anaemic iron deficient individuals: protocol for an individual patient data meta-analysis (IPMA). PROSPERO 2020 CRD42020191739 Available from:
https://www.crd.york.ac.uk/prospero/display_record.php?ID=CRD42020191739

Review question [1 change]

In individuals with a serum ferritin < 20 ug/L does iron supplementation improve fatigue and physical capacity

Searches [1 change]

All Potential studies published in the literature will be sought from systematic searches (from inception to present) conducted in PubMed, MEDLINE, and Cochrane Library databases using the following search terms: iron, dietary iron, ferritin, supplement, anaemia, athletic performance, physical capacity, athletes, physical endurance. Studies will then be initially screened by a minimum of 2 trial authors, and all non-human studies will be immediately eliminated. Please note, no restriction will be placed on the searchers due to language (i.e. studies of any language may be included) or publication date. Studies will then be further screened (by a minimum of 2 authors) in accordance with the predetermined inclusion and exclusion criteria (i.e. full text screening).

Search strategy

https://www.crd.york.ac.uk/PROSPEROFILES/191739_STRATEGY_20201120.pdf

Types of study to be included

There will be no restriction on the type of study design eligible for inclusion.

Condition or domain being studied

This individual patient data meta analysis (IPDMA) will be investigating the efficacy of iron repletion therapies on physical capacity and fatigue in non-anaemia iron deficient (IDNA) individuals.

Participants/population [2 changes]

Inclusion criteria

(1) Individuals aged between 12-45 years old

(2) Individuals who are iron deficient (serum ferritin < 20ug/L)

(3) Individuals who are not anaemic (haemoglobin concentration greater than 130 g/L for men and greater than 120 g/L for non-pregnant women)

Exclusion criteria

(1) Individuals (Females) who are pregnant or lactating

(2) Individuals with hemochromatosis or other relating genetic diseases

(3) Individuals with other underlying medical conditions requiring hospitalisation (for example, heart failure patients)

(4) Studies involving animals

Intervention(s), exposure(s)

Inclusion criteria

(1) Both nutritional supplementation and single therapy interventions will be considered. These include; (a) diet manipulation; (b) oral iron supplement; (c) intravenous iron therapy; (d) intramuscular iron therapy

Comparator(s)/control

(1) No intervention

(2) Placebo

(3) Other active therapies (i.e. diet manipulation)

Context

Cochrane recently published a comparative paper highlighting the differences between using summary aggregate data and individual patient data for meta-analyses (Smith et al., 2016). Using 39 different meta analyses for comparison, it was found that 4 out of 5 times, similar conclusions can be drawn. Therefore, it is recommended that an aggregate data meta-analysis should be conducted prior to completing an IPMA. It was further concluded that should any shortcomings exist with an aggregate data meta-analysis, then an IPMA should be considered whilst remembering the extra work involved (Smith et al., 2016). The published aggregate data reviews specific to the efficacy of iron supplementation on performance in IDNA individuals has conflicting outcomes (Burden et al., 2015; Houston et al., 2018; Rubeor et al., 2018; Miles et al., 2019). Indeed, no clear consensus has emerged from these reviews, with the latest Cochrane paper stating that substantial heterogeneity significantly impacted the analysis and results. Therefore, an IPMA that narrows down the scope of included participants is necessary to resolve the current ambiguity in the literature. Hence, our inclusion and exclusion criteria reflect this accordingly.

Main outcome(s)

(1) Measures of quality of life as defined by the trial authors

(2) Measures of work capacity as defined by the trial authors.

(3) Measures of fatigue as defined by the trial authors

Measures of effect

Effect sizes and standardised mean difference (SMD) will be used for continuous outcomes. Effect sizes and Odds ratios will be utilised for dichotomous outcomes.

Additional outcome(s) [1 change]

(1) Other measures of physical capacity (e.g. time trial times, time to exhaustion)

(2) Mean change in haemoglobin concentration

(3) Mean change in total haemoglobin mass

(4) Mean change in iron markers of the blood (e.g. serum ferritin)

(5) Adverse events

Measures of effect

Effect sizes and standardised mean difference (SMD) will be used for continuous outcomes. Effect sizes and Odds ratios (OR) will be utilised for dichotomous outcomes.

Data extraction (selection and coding)

All Potential studies published in the literature will be sought from systematic searches conducted in PubMed, MEDLINE, and Cochrane databases using the following search terms: iron, dietary iron, ferritin, supplement, anaemia, athletic performance, physical capacity, athletes, physical endurance. Studies will then be initially screened by a minimum of 2 trial authors, and all non-human studies will be immediately eliminated. Studies will be further screened (by a minimum of 2 authors) in accordance with the predetermined inclusion and exclusion criteria (i.e. full text screening).

Authors of included papers will be contacted individually and requested to provide all data sets. However, only data concerning the specific individual patients who meet the inclusion/exclusion criteria will be included in the analysis. Datasets provided by authors will be combined into a single SPSS file. Data from this single file will then be transcribed in the Cochrane Review Manager software (RevMan 5.0).

Risk of bias (quality) assessment

The internal validity of the included studies will be assessed using the Cochrane Collaboration Risk of Bias tool (Higgins et al., 2011). This tool assesses bias of studies according to sequence generation, allocation concealment, blinding, incomplete outcome data (e.g. dropouts and withdrawals) and selective outcome reporting. Evidence will be graded according to strength and will be categorised as either “very low”, “low”, “moderate” or “high” using the Grading of Recommendations Assessment, Development and Evaluation Methodology (Higgins et al., 2011). Accordingly, RevMan will be used to present findings graphically to highlight any major biases found within the individual patient data provided to us by the authors of individual studies.

Strategy for data synthesis [1 change]

As mentioned above, authors of included papers will be contacted individually and requested to provide all data sets. However, only data concerning the specific individual patients who meet the inclusion/exclusion criteria will be included in the analysis. Datasets provided by authors will be combined into a single SPSS file. Data from this single file will then be transcribed in the Cochrane Review Manager software (RevMan 5.0) to calculate the pooled estimates of the effect and the 95% confidence intervals for the pooled effect for each of the outcomes, using a random effects model (assuming that the data is homogeneous in nature). Continuous data will be expressed as a standardised mean difference (SMD), whereas dichotomous data will be expressed in Odds Ratio's (OR). Further analysis within RevMan will provide heterogeneity levels for each of the subgroups across the different studies. Indeed, statistical heterogeneity of the data will be quantified using the I^2 test. Should heterogeneity be detected, then further analysis (i.e. subgroup analysis or sensitivity analysis) will be conducted.

Analysis of subgroups or subsets [1 change]

(1) Sex (male vs. female)

(2) Serum ferritin cut off values (<30ug/L; <20ug/L; <15ug/L)

(3) Exercise capacity (high vs. lower)

(4) Types of supplementation strategy (oral vs. intravenous vs. intramuscular)

(5) Duration and dosage of the supplementation used

(6) Trials at low-risk vs. high-risk of bias (sensisitivty analysis)

(7) Conflicts of interest (i.e. sponsored studies vs. non-sponsored studies)

(8) Time to follow up

Contact details for further information

Cory Dugan

cory.dugan@research.uwa.edu.au

Organisational affiliation of the review

The University of Western Australia

https://www.uwa.edu.au/

Review team members and their organisational affiliations

Mr Cory Dugan. The University of Western Australia

Professor Toby Richards. The University of Western Australia

Assistant/Associate Professor Peter Peeling. The University of Western Australia

Ms Giulianna D’Aulerio. The University of Western Australia

Dr Lawrence Best. University College London

Type and method of review

Individual patient data (IPD) meta-analysis, Systematic review

Anticipated or actual start date

01 January 2021

Anticipated completion date

01 January 2023

Funding sources/sponsors

The University of Western Australia

Grant number(s)

State the funder, grant or award number and the date of award

UWA RTP Scholarship. Awarded 05/01/2020.

Conflicts of interest

Language

English

Country

Australia, England

Stage of review

Review Ongoing

Subject index terms status

Subject indexing assigned by CRD

Subject index terms

Dietary Supplements; Ferritins; Humans; Iron; Iron Compounds

Date of registration in PROSPERO

10 December 2020

Date of first submission

20 November 2020

Stage of review at time of this submission

The review has not started

Stage	Started	Completed
Preliminary searches	No	No
Piloting of the study selection process	No	No
Formal screening of search results against eligibility criteria	No	No
Data extraction	No	No
Risk of bias (quality) assessment	No	No
Data analysis	No	No

Revision note

Inclusion criteria age has changed to allow for a greater inclusion of studies. Inclusion criteria threshold for iron deficiency changed in accordance with guidelines from previous research (Rubeor et al., 2018).

The record owner confirms that the information they have supplied for this submission is accurate and complete and they understand that deliberate provision of inaccurate information or omission of data may be construed as scientific misconduct.

The record owner confirms that they will update the status of the review when it is completed and will add publication details in due course.

Versions

10 December 2020

04 May 2022

30 June 2022
