# Supplementary material for: Efficacy of iron supplementation on physical capacity in non-anaemic iron-deficient individuals: protocol for an individual patient data meta-analysis
Source: Syst Rev. 2024 Jul 15;13:182. doi: 10.1186/s13643-024-02559-4 (PMC11247796; doi:10.1186/s13643-024-02559-4)
Supplement: Supplementary file 2 — Additional file 2. Draft search strategy. [file 13643_2024_2559_MOESM2_ESM.pdf]

OVID Search Strategy (DRAFT ONLY)

1. Anemia, Iron-Deficiency/ or Iron, Dietary/

2. (iron adj4 supplement*).mp. [mp=title, abstract, original title, name of substance word, subject heading word, floating sub-heading word, keyword heading word, organism supplementary concept word, protocol supplementary concept word, rare disease supplementary concept word, unique identifier, synonyms]

3. 1 or 2

4. ("iron therapy" or "iron treatment" or "iron deficiency treatment").mp. [mp=title, abstract, original title, name of substance word, subject heading word, floating sub-heading word, keyword heading word, organism supplementary concept word, protocol supplementary concept word, rare disease supplementary concept word, unique identifier, synonyms]

5. 3 or 4

6. ("intravenous iron use" or "intravenous iron injection" or "intravenous iron infusion").mp. [mp=title, abstract, original title, name of substance word, subject heading word, floating sub-heading word, keyword heading word, organism supplementary concept word, protocol supplementary concept word, rare disease supplementary concept word, unique identifier, synonyms]

7. 5 or 6

8. Physical Fitness/de, ph [Drug Effects, Physiology]

9. Physical Endurance/de, ph [Drug Effects, Physiology]

10. 8 or 9

11. Oxygen Consumption/de, ph [Drug Effects, Physiology]

12. 10 or 11

13. Exercise Test/de [Drug Effects]

14. 12 or 13

15. 7 and 14
